# Assessing the knowledge, attitude and practice of family planning among women living in the Mbouda health district, Cameroon

**DOI:** 10.1186/s12978-015-0085-9

**Published:** 2015-10-09

**Authors:** Jobert Richie N. Nansseu, Emmanuel Choffor Nchinda, Jean-Claude Katte, Fatima M. Nchagnouot, Guylaine D. Nguetsa

**Affiliations:** Department of Public Health, Faculty of Medicine and Biomedical Sciences, University of Yaoundé I, PO Box 1364, Yaoundé, Cameroon; Sickle Cell Disease Unit, Mother and Child Centre of the Chantal Biya Foundation, Yaoundé, Cameroon; Department of ENT and Head and Neck Surgery, Faculty of Medicine and Biomedical Sciences, University of Yaoundé I, Yaoundé, Cameroon; Diabetes and Hypertension Treatment Centre, Bafoussam Regional Hospital, Bafoussam, Cameroon; Pediatric Unit, Bertoua Regional Hospital, Bertoua, Cameroon

**Keywords:** Knowledge, Attitude, Practice, Contraception, Family planning, Cameroon

## Abstract

**Background and objective:**

Promotion of family planning has been shown to reduce poverty, hunger, maternal and infant mortality, and contribute to women’s empowerment. But many resource-limited countries still have very low rates of contraceptive use. The present study aimed to assess the knowledge, attitude and practice of family planning among women living in a resource-poor rural setting.

**Methods:**

We conducted a cross-sectional study in January 2010 in the Mbouda Health District, Cameroon. After a multistage random selection, 120 households were selected. Participants were women aged at least 15 years old, sexually active, and who volunteered to participate in the study. Data were collected during an anonymous interview using a structured pre-tested questionnaire.

**Results:**

A total of 101 women were enrolled, their ages ranging from 18–58 years with a mean of 31.7 ± 8.8 years. Ninety-six percent of these women had already heard about family planning. Almost all respondents (98 %) were aware of at least one contraceptive method, the most cited being the male condom (96 %), the safe period (86.1 %), injectables (76.2 %) and oral pills (75.2 %), Sixty-six women (65.3 %) were currently practicing at least one contraceptive method, and the three prevailing methods used were: the safe period (50 %), the male condom (34.8 %), and injectables (12.1 %). The main reasons precluding women from practicing contraception were lack of knowledge (31.4 %), uselessness (31.4 %) and unbearable side effects (8.6 %). Fourteen of these women (42.4 %) expressed the willingness to start practicing contraception if they received more information about the subject. Decision on the number of children to have was made by both the man and the woman in 59.5 % of cases. The practice of contraception had been decided by the couple in 39.6 % of cases, and 9.4 % of men were not aware that their wives were currently practicing contraception.

**Conclusion:**

Although the level of awareness about family planning and contraceptive methods is quite satisfactory, the level of contraceptive use is not optimal in our setting. Consequently, more adapted educational and counseling interventions should be undertaken among women, and family planning messages directed to men need to be included too.

## Introduction

There is convincing evidence that poverty incidence is always higher among larger households. Indeed, Orbeta A. [1] figured out an enduring positive association between family size and poverty incidence and severity. He also showed how a large family size creates the conditions leading to greater poverty through its negative impact on household savings, labor force participation, and earnings of parents, as well as on the human capital investment in children [1]. Besides, we read from Renjhen et al. [2] that uncontrolled population growth is recognized as the single most important impediment to national development. Therefore, the promotion of family planning, especially in countries with high birth rates, has the potential to reduce poverty and hunger, and avert 32 % of all maternal deaths and nearly 10 % of childhood deaths [3]. It may also substancially contribute to women’s empowerment, achievement of universal primary schooling, and long-term environmental sustainability [3].

Programmes to promote family planning in developing countries began in the 1960s, with the number of countries with official policies to support family planning rising from only two in 1960–74 by 1975 and 115 by 1996 [4]. Between 1960 and 2000, the proportion of married women in developing regions using contraception increased from less than 10 to about 60 %, though with huge variations from one area to another [5]. However, many of today’s poorest countries, mainly in sub-Saharan Africa (SSA), still have high fertility and high unmet needs for family planning, and their populations are projected to double in the next few decades [3]. Moreover, in most African countries, high fertility and rapid population growth represent a bigger threat to achievement of the Millennium Development Goals than infectious diseases such as HIV/AIDS [3].

In 1986, the Cameroonian Government officially clarified its position about promoting the practice of family planning in the country, though there is dearth of information in this regard all over the country. Data from the Demographic and Health Survey (DHS) revealed a 26 and 24 % prevalence of contraceptive uptake respectively in 2004 and 2011 [6], hence a very low number of women using contraception. The present study aimed at assessing the knowledge, attitude and practice of family planning among women of a Cameroonian health district, specifically in a rural setting, and investigating male participation in family planning from the point of view of their female partner.

## Methods

### Study design and participants

This was a cross-sectional study conducted in January 2010 in the Mbouda Health District, Cameroon. This is one of the 19 health districts of the West Region of Cameroon, extending over 455 km^2^, and comprising nearly 227,448 inhabitants in 2010. The district is divided into 12 health areas among which 4 were randomly selected for recruitment: the Mbouda-West, Mbouda-North, Bafounda, and Toumaka health areas. In each of these zones, we randomly selected three neighborhoods among each of which ten households to be visited were subsequently retained, hence a total of 120 households. We were helped in this process by the sanitary authorities of the district alongside the heads of each selected health area with whom we visited each selected household in their respective zones.

Participants were women aged at least 15 years old, irrespective of their marital status and educational level, currently residing in the district, and who volunteered to take part in the study. Data were collected during an interview using a structured pre-tested questionnaire recording socio-demographic background (age, level of education, occupation, religion, marital status, type of household, family size, birth interval, number of children per bedrooms, and number of meals per day), knowledge on family planning, current method of contraception used, and the level of implication of the husband. Nineteen of the 120 households visited were not included in the study because the woman was absent when the investigator visited (10), or because of refusal to participate (9).

### End-point definitions

We defined family planning as all the measures undertaken to limit and space births. In this regard, “limitation of births” referred to controlling the number of births, whereas “stopping births” meant ending deliveries by a definite or permanent method. On another hand, contraception was defined as prevention of conception, i.e. any method used not to become pregnant.

### Statistical analysis

Data were coded, entered and analyzed using Epi info version 3.3.2 (Center for Disease Control, Atlanta, USA). Results are presented as mean ± standard deviation (SD) for quantitative variables, and count (percentage) for qualitative variables. Qualitative variable comparisons used the chi-square test or equivalents where appropriate. Odds ratios (OR) with 95 % confidence intervals were used to examine the impact of contraception on various outcomes. Results were considered statistically significant if *p value* < 0.05.

## Ethical considerations

Before carrying-out this study, authorizations were obtained from the administrative and sanitary authorities of the study site, acting as ethical review board. Participants were informed of the various aspects of the study, and were anonymously included in the survey after they had signed the informed consent form. The study was conducted in accordance with the revised Helsinki Declaration.

## Results

We enrolled a total of 101 participants. Table 1 depicts the main socio-demographic characteristics of the study population. Ages varied between 18 and 58 years, with a mean of 31.7 ± 8.8 years. The most encountered age group was 20–24 years (21.8 %, see Table 1). Fifty nine women (58.4 %) had a secondary educational level, 51 (50.5 %) were housewives, and nearly two thirds of respondents (65.3 %) belonged to monogamic households (see Table 1).

**Table 1:** Socio-demographic characteristics of the study population

Characteristic	Number (*N* = 101)	Percentage (%)
Age (years)		
<20	3	2,9
20–24	22	21,8
25–29	19	18,8
30–34	19	18,8
35–39	20	19,8
40–44	9	8,9
≥45	9	8,9
Educational level		
Never went to school	4	4,0
Primary	37	36,6
Secondary	59	58,4
University	1	1,0
Profession		
Housewife	51	50,5
Farmer	18	17,8
Trader	21	20,8
Civil servant	11	10,9
Type of household		
Single parent	4	4,0
Monogamic	66	65,3
Polygamic	31	30,7

The large majority of women (96 %) had already heard about family planning, nearly half of them (58.8 %) during educative sessions as part of antenatal care delivered in health facilities, but only 4 women (4.1 %) gave the right definition of family planning (see Table 2). Eighty women (79.2 %) had never visited a family planning centre. About two thirds of participants (60.3 %) had already heard about contraception, the exact definition being given by 38 (62.5 %) of them (see Table 2).

**Table 2:** Knowledge and practice of family planning

	Number	Percentage (%)
Source of information (*N* = 97)		
Health centers	57	58.8
Medias	28	28.9
Entourage	12	12.3
Family planning is: (*N* = 97)		
Limitation of births	28	28.9
Spacing of births	35	36.1
Stopping births	11	11.3
Limitation and spacing of births	4	4.1
No idea	19	19.6
Ever visited a family planning center (*N* = 101): yes	21	20.8
Ever heard about contraception (*N* = 101): yes	61	60.3
Contraception is: (*N* = 61)		
Prevention of conception (i.e. getting pregnant)	38	62.3
No idea	23	37.7
Contraceptive methods known (*N* = 101)		
Male condom	97	96
Female condom	36	35.6
Safe period	87	86.1
Injectables	77	76.2
Oral pills	76	75.2
Implants (Norplant)	57	56.4
Coitus interruptus (withdrawal)	42	41.5
Intrauterine device (IUD)	40	39.6
Female sterilization	20	19.8
Maternal breastfeeding	6	5.9
Temperature	5	4.9
Abstinence	4	3.9
Spermicides	3	2.9
Male sterilization	3	2.9
Diaphragm	1	0.9
Practice of contraception (*N* = 101): yes	66	65.3
Reasons precluding women to practice contraception (*N* = 35)		
Unbearable side effects	3	8.6
May lead to cancers	2	5.7
Useless	11	31.4
Ignorance/lack of knowledge	11	31.4
Other reasons	8	22.9
Birth interval (years) *N* = 101		
1	6	6.0
2	33	32.7
3	27	26.7
>3	17	16.8
Missing data	18	17.8
Number of children per bedroom (*N* = 101)		
≤2	55	54.5
>2	46	45.5
Number of meals per day (*N* = 101)		
<3	42	41.6
At least 3	59	58.4

Table 2 spells out among others, the different methods of contraception known by our respondents. The most cited ones were: the male condom (96 %), the safe period (86.1 %), injectables (76.2 %) and oral pills (75.2 %). Five point nine percent of women cited one method, 8.9 % two methods, 7.9 % three methods, 5.9 % four methods, 15.8 % five methods, and 53.4 % of women cited more than 5 methods, but 2 % of participants were not aware of any contraceptive method (see Table 2).

**Fig. 1:**
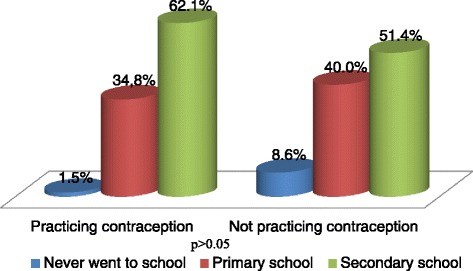
Comparison between women practicing contraception (*on the left*) and those not practicing contraception (*on the right*) with regard to educational level; no influence of the level of education on the practice or not of contraception (*p* > 0.05)

**Fig. 2:**
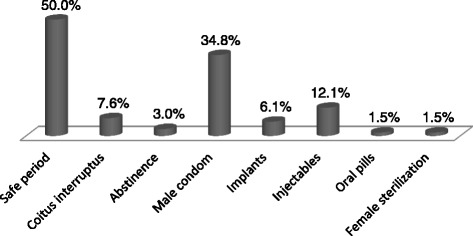
Different contraceptive methods used by women currently on contraception. (The overall percentage >100 % because one woman could be using a combination of two or several contraceptive methods)

Sixty-six women (65.3 %) were currently practicing at least one contraceptive method. Figure 1 presents a comparison between women practicing contraception or not with regard to their level of education. There was no relation between these two variables (*p* > 0.05), as well as with age, marital status and occupation (all *p* values > 0.05). Among the women not practicing contraception, 14 (42.4 %) expressed the willingness to start doing so in the near future if they received more information about the subject. Figure 2 depicts the different methods used by women currently practicing contraception. The three main methods used were: the safe period (50 %), the male condom (34.8 %), and injectables (12.1 %). More than one method could be used by the same participant, the prevailing combination being safe period + male condom.

Among the women actively practicing contraception, 18 (27.7 %) were not satisfied with the current method used, the reasons being: ineffectiveness (55.6 %), side effects (22.2 %), and difficult usage (22.2 %). Of these women, 8 (44.4 %) were on the safe period, 7 (38.9 %) used the male condom, 2 (11.1 %) on injectables, and 1 (5.6 %) on the association safe period + male condom. Ten women (15.2 %) practicing contraception had undesirably become pregnant sometime in the past, among which 7 (70 %) used the safe period, 1 (10 %) the male condom, 1 (10 %) the injectables, and 1 (10 %) used the combination safe period + male condom.

**Fig. 3:**
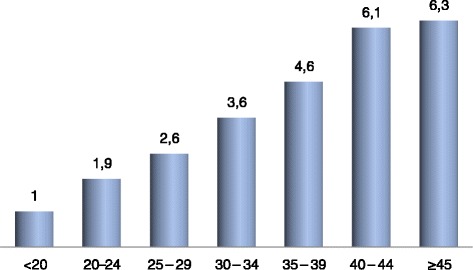
Average number of children with respect to age-groups. Intriguingly, women seem to continue delivering even into advanced ages (≥40 years)

The number of persons in the household varied from 2–40, with a mean of 7.3 ± 5.4 persons. The number of children per household varied between 1 and 18 with a mean of 4.5 ± 3.1 children. The number of children per woman ranged from 1–9, with a mean of 3.7 ± 2.1 children. Figure 3 is representative of the average number of children per age-group of the women interviewed. Women aged 40–44 had 6.1 children compared to women aged 35–39 who had 4.6 children, hence the inference that around or after 40 years old, the women of our study population continue to give birth. The dominating birth interval was 2 years (39.8 %); with the mean equal to 2.8 ± 0.9 years.

In households not under family planning, there was a significant 3.2 increased likelihood to have less than three meals per day (95 % CI: 1.3-8.2; *p* < 0.05). Children living in households under family planning had a 1.2 fold increased risk of being more than 2 per bedroom, though statistically non-significant (95 % CI: 0.5-2.7; *p* > 0.05).

Decision on the number of children to have was made by both the man and the woman in 59.5 % of cases, whereas this discussion had never existed in 23 % of cases. The practice of contraception had been decided by the couple in 39.6 % of cases, only by the woman in 40 % of cases, and only by the man in 23.1 % of cases. Nine point four percent of men were not aware that their wives were currently practicing contraception.

## Discussion

The introduction of modern contraceptive techniques over the recent decades and the increasing availability of safer and more effective methods of preventing pregnancy have permitted people around the world to exercise their choice, make responsible decisions with respect to their reproduction and enjoy the benefits of family planning [7]. The current prevalence of contraceptive use is thus approaching 60 % worldwide, and in the less developed countries, almost 53 % of couples are using some form of contraception [4, 5, 8]. Results from the present study, conducted in a typical rural SSA setting, reveal a 65.3 % prevalence of contraception practice, highly superior to the 24 % reported by the 2011 Cameroon DHS, and nearly two times the specific prevalence in the West Region of the country (33.7 %) [6]. Besides, the overall prevalence of contraception practice in Cameroon has not changed from 2004–2011: 26 and 24 % respectively [6], questioning therefore the efforts that have been made so far to vulgarize such a practice around the country during the last decade. Presumably, a lot of work still needs to be done in this regard. For instance, more educational and counseling interventions should be undertaken everywhere (media, churches, schools, health centers among others), with special emphasis on the advantages of modern contraceptives.

Ninety six percent of our respondents had already heard about family planning, the main source of information being the health personnel (58.8 %). This finding corroborates that of Ghazal-Aswad et al. [7] where primary health care physicians were cited as main source of information. Contrariwise, school and friends/relatives [9, 10], or media [2] were the main sources of information reported by other authors. Therefore, our primary health care providers have a major role to play in improving the women’s knowledge of family planning and awareness of different contraceptive methods available alongside their respective advantages and inconveniences. In this regard, primary health care providers’ knowledge and skills must be continuously enhanced and reinforced to deliver the right and sound advice about contraception. Besides, although the large majority of women had heard about family planning, very few of them (4.1 %) knew exactly what it refers to, and 60.3 % had heard about contraception before. This finding raises the issue of the content of information transmitted to women on family planning, contraception and contraceptive usage, stressing perhaps the need to adapt the message to be passed, may be by delivering it in appropriate words and in the women’s mother tongues. We have seen indeed that the large majority of our women had a secondary or less level of education, presumably hindering their capacity of understanding if scientific or complicated words are used.

Though some women had never heard about contraception (39.7 %), we found that, after the investigator explained what it refers to, 98 % of participants were aware of at least one contraceptive method, with more than half of them (53.4 %) being able to cite more than 5 contraceptive methods. The most cited methods were the modern ones (mainly condom, injectables, pills, implants, intrauterine device, and sterilization), followed by the traditional ones (mostly the safe period and coitus interruptus). Concurring our results, the 2011 Cameroon DHS revealed that 94 % of women were able to cite at least one contraceptive method, the most cited being the modern ones (the male condom at first as in our study) [6]. Likewise, Omo-Aghoja et al. [9] reported, in a rural Nigerian setting, a 92.3 % level of contraceptive awareness, the most widely known contraceptive methods being injectables, condoms and pills. It is worth noticing that the level of knowledge of contraceptive methods has increased over time in Cameroon: 73 % in 1991, 81 % in 1998, 90 % in 2004, and 94 % in 2011 [6].

However, this evolution contrasts with that of the practice of contraception which remains very low: 16 % in 1991, 19 % in 1998, 26 % in 2004 and 24 % in 2011 [6]. There is thus a large gap between contraceptive knowledge, approval and practice in our context. In our study, 39.7 % of women were not currently practicing contraception, and the prevailing reasons precluding this practice were lack of information (31.4 %), uselessness (31.4 %), and fear of side effects (14.3 %). In Nigeria, the factors associated with low contraceptive usage were: poor level of training and ineffective conveyance of relevant information to women by health personnel, low literacy levels, extremes of reproductive age and extremes of parity, fear of side effects, lack of knowledge and lack of spousal consent [9]. Other important factors affecting the use of contraceptive methods are unsatisfactory sexual life, inaccessibility of contraceptives, socioeconomic status, cultural background and religious belief [2, 7, 11, 12]. These barriers have therefore to be taken into consideration when carrying the education and communication programmes in order to enhance contraception uptake and usage in our settings, especially that of modern contraceptives. For instance, 14 out of 35 women currently not practicing contraception expressed the willingness to do so if they received more information. Besides, more studies dedicated at a thorough investigation of the different reasons pertaining the nonuse of contraception and how these can be addressed, are urgently warranted in our milieu.

The dominating contraceptive methods used were the traditional ones (safe period, coitus interruptus, and abstinence: 60.6 %), followed by modern methods (male condom, pills, injectables, implants and female sterilization: 39.4 %), corroborating Ghazal-Aswad et al. [7] findings. By contrast, the 2011 Cameroon DHS showed that modern methods were more used than traditional ones [6]. In the same line, results from Omo-Aghoja et al. [9] showed that modern methods were those mostly used by women of the Amukpe community, Nigeria. Unfortunately, we did not search for the reasons forcing our women to choose one contraceptive method instead of another one. It is therefore difficult to explain why the traditional methods, which by the way are less reliable and need more efforts, outweighed the modern ones in our study. We can guess that, due to low educational and socioeconomic levels, cultural and religious beliefs, and lack of roads, modern contraceptives are perhaps unavailable, inaccessible or unaffordable. Studies targeting the cost-benefit and acceptance of each of the contraceptive methods should be conducted to address the right message during educative sessions in our settings. These sessions must focus on the advantages of modern contraceptives methods to demystify them and increase thereby their uptake.

Regrettably, the cross-sectional design of the present study precluded us from further investigations. Additionally, the self-reporting of information could have introduced some bias. Nonetheless, interviews were anonymous and confidential to make sure that none of the participants could feel uncomfortable while answering the questions. Although our findings cannot be generalized to the entire Cameroonian female population, we used a rigorous multistage random sampling method to recruit our respondents, enabling us to generalize our results to the entire female population of the Mbouda health district, though our sample size could be seen as relatively low.

While investigating male participation in family planning and contraceptive use, we found that there is a lack of male–female communication regarding the number of children to have and the practice of contraception, in line with Kaida et al. findings in Mpigi district, Uganda [13]. On the contrary, Lasee and Becker [14] reported that husband-wife communication about family planning was effective in 82 % of their couples. In the large majority of SSA settings, men have suffered from limited involvement in either receiving or providing reproductive health information, hence they are generally uninformed about contraceptive methods [13, 15]. Additionally, it has been bolstered that women who did not discuss family planning with their male partner had a 2.8-fold increased risk for an unplanned pregnancy [16]. There is body of evidence that partner communication about family planning is associated with and is often essential to increase levels of knowledge, improve attitudes, and enhance the use of family planning methods [13–15, 17, 18]. Consequently, it clearly appears of urgent need to broaden the scope of family planning programmes by including family planning messages directed to men.

## Conclusion

Although the level of awareness about family planning and contraceptive methods is quite satisfactory, the level of contraceptive uptake is not optimal in our setting. In this regard, more adapted educational and counseling interventions should be undertaken among women, and family planning messages directed to men need to be included too. Furthermore, primary health care providers’ knowledge and skills have to be continuously enhanced and strengthened to deliver the right and sound advice about family planning and contraception. Moreover, more studies dedicated at a thorough investigation of the different reasons pertaining the nonuse of contraception and how these can be addressed, are warranted. Additionally, future studies assessing the cost-benefit and acceptance of each of the contraceptive methods should be conducted to address the right message during educative sessions.
